# Effect of plastic and seaweed shelters on the skin microbiome of lumpfish *Cyclopterus lumpus* used as cleaner fish in aquaculture pens

**DOI:** 10.1371/journal.pone.0322261

**Published:** 2025-09-03

**Authors:** Ása Jacobsen, Agnes Mols Mortensen, Kirstin Eliasen, Elin Egholm, Marner Nolsøe, Ása Johannesen

**Affiliations:** 1 Department of Biotechnology, Firum PF, Tórshavn, The Faroe Islands; 2 Department of Research, Tari Spf., Fámjin, The Faroe Islands; 3 Department of Fish Health, Firum PF, Hvalvík, The Faroe Islands; 4 Quality Department, Bakkafrost, Glyvrar, The Faroe Islands; 5 Department of Ecology, Firum PF, Hvalvík, The Faroe Islands; Hokkaido University, JAPAN

## Abstract

Atlantic salmon (*Salmo salar*) aquaculture is a major industry in several countries worldwide and a growing enterprise in others. One of the main challenges the industry faces is infestations with the parasitic copepod *Lepeoptheirus salmonis*, or salmon lice. Several different chemical and mechanical methods are available for alleviating the problem, but often at cost to salmon welfare and/or the environment. In some regions cleaner fish have been introduced to farming facilities as an environmentally and salmon welfare friendly option for reducing sea lice infestations. In some North Atlantic countries, lumpfish (*Cyclopterus lumpus*) are being used as cleaner fish. However, poor welfare and high mortality rates of lumpfish in salmon farming are frequent issues, and the need to improve lumpfish welfare is great. One adaptation for salmon farms is to provide the lumpfish with shelters to meet their need to rest and hide. Plastic shelters are the most widely used form, but seaweed shelters have more recently also been applied as a more natural solution. This project investigated the potential effect of seaweed and plastic shelters on the skin and gill microbiome of lumpfish and any potential correlation to their welfare. In an experimental setup in a commercial salmon farming facility, lumpfish from pens with either plastic or seaweed shelters were sampled over a period of approximately three months. The results showed that the bacterial communities on the two shelter types were significantly different and fewer potentially pathogenic bacteria dominated the skin microbiome of lumpfish living with seaweed shelters than of those living with plastic shelters. No differences were detected in the welfare of the lumpfish and further investigations are needed to clarify any potential implications of the differences detected in the skin microbiome of lumpfish including responses to stressful conditions.

## Introduction

Atlantic salmon (*Salmo salar*) aquaculture is a major industry in several North Atlantic countries. Although highly successful, there are many challenges in the industry and one of the main challenges is infestations of salmon lice (*Lepeoptheirus salmonis*) [1], an ectoparasitic copepod that feeds on the mucus, skin and blood of its host. To mitigate this problem various chemical and mechanical solutions have been and are currently being used with various effectiveness. However, there are several drawbacks of these approaches such as diminishing effects of medical treatments over time, negative environmental impact and/or substantial salmon welfare issues [2].

To avoid these concerns, cleaner fish have been introduced as a biological control to reduce sea lice infestations without adverse effects on salmon welfare or the environment. In the Faroe Islands and Norway, lumpfish (*Cyclopterus lumpus*) are being used as cleaner fish in the aquaculture industry and although this approach has shown to be relatively effective [3–5], lumpfish are a new species in aquaculture and have quite different abilities and requirements than salmon. This has resulted in challenges concerning their poor welfare and mortality rates are high due to a range of unresolved issues [6,7]. A major problem is the high frequency of bacterial infections by known pathogens such as *Tenacibaculum* spp, *Pseudomonas* spp, *Aeromonas* spp. and *Vibrio* spp. [8], which are more likely to occur due to lumpfish already being weakened by other factors [9].

The bacterial communities are part of the microbiome, which encompasses all microbial communities as well as their activity and interactions in any defined habitat with distinct micro-environmental properties [10]. The fish mucosal barriers and their microbiomes contain both a range of immunogenic compounds as well microbial communities, and in healthy fish the immune system is able to maintain homeostasis without either infections or excessive immune responses [11,12]. At the same time, the fish mucus microbiome is affected by various factors such as the host genetics, environment and diet [11,12]. One example of how the environment can influence the fish microbiome is the impact of sea anemones on the skin microbiome of fish that live in close contact with them [13]. On the other hand, several studies have found that bacterial communities in the fish skin microbiomes can show a limited correlation to the bacterial communities in the surrounding water [14,15].

Because lumpfish are not adept swimmers and lack a swim bladder, salmon pens with lumpfish are usually equipped with shelters. These built environments are essential for lumpfish welfare as they offer the possibility to hide, rest, and thrive in salmon farming facilities [16]. The shelters have usually been constructed of plastic, but in recent years natural seaweed shelters have gained increasing awareness as a more natural alternative. It is well documented that seaweeds contain a range of antibacterial components [17], which might affect the microbiome of fish living in near contact with them. Here, we describe a study performed to investigate the potential effect plastic and seaweed shelters have on skin microbiome and health of lumpfish in aquaculture pens. The hypothesis is that seaweed shelters reduce the prevalence of pathogenic bacteria in the microbiome of lumpfish living among them compared to the microbiome of lumpfish living with plastic shelters. Studies of potential impacts of built environments on their inhabitants have mainly focused on the human health in terrestrial environments [18]. And, while several studies have investigated fish microbiomes in land-based facilities [19,20], much less attention has been paid to potential impacts of built environments on microbiomes and health of fish living in the ocean. Hence, the knowledge gained from this study can be important for all fish species used as cleaner fish in the aquaculture industry and that require shelters, in addition to other fish species living in marine built environments.

In addition, knowledge about lumpfish mucosal or skin microbiomes is limited. Therefore, the results from this study will provide valuable contributions to an increased understanding, contributing to the effort of improving lumpfish welfare in aquaculture facilities and to any potential future investigations into circumstances resulting in detrimental infections.

## Materials and methods

### Ethics statement

The Firum Ethical Committee (Internal Review Board) waived ethical review for this study and did not require additional approval. All handling and sacrifice of lumpfish were part of the operational procedures of the aquaculture farm site, which followed national guidelines for routine welfare assessment and farm site management.

### Field site and setup

**Fig 1:**
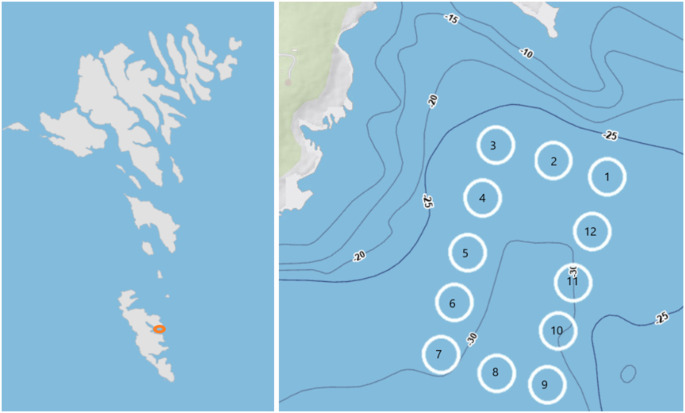
Experimental site. Geographic location of fieldwork on (a) The Faroe Islands and (b) experimental setup in Froðba. Map data is from the Faroese Environment Agency ©Umhvørvisstovan: kort.foroyakort.fo. Data from the Faroese Environment Agency includes depth curves: dýpdarkurvar 5m. Salmon farming pens no. 4, 7, and 12 contained seaweed shelters, pens no. 1, 3, and 8 contained plastic shelters and the remaining pens were not used in this study.

The experiment took place in the Northeast Atlantic archipelago, the Faroe Islands (Fig 1a), at a salmon farming facility owned and operated by Bakkafrost. The farming site was located in Froðba in a coastal area with good water exchange [21] providing basis for equal environmental conditions in the different pens. The farming site contained 12 salmon farming pens (~150m circumference and ~15m deep) of which the project had access to sampling and measurements in six; three containing plastic shelters and three containing seaweed shelters (Fig 1b). No lumpfish were added to pens without shelters.

During the study period, from medio April to early August 2023, the temperature increased from approximately 7.4 to 10.4 degrees Celsius and oxygen levels were between 8.3 and 9.9 mg/L (S1 Data). Salinity was stable at 35 ppm. Between approximately 85,000 and 99,000 salmon were deployed to each of the six pens in December 2022 and the average weight for the salmon deployed to each pen was between 183 g and 397 g. In the study period, the average weights of salmon in each pen were between 1.06 kg and 1.39 kg in early May and between 1.79 kg and 2.28 kg in early July. The salmon had no history of disease and no treatments, medical nor mechanical, were carried out while the experiment was ongoing. Salmon feeding was controlled with the use of feed cameras and was delivered using automatic feed spreaders while the lumpfish were fed with 3 mm lumpfish feed from Havsbrún once a day. Lumpfish (approximately 25g) were added to the six pens in April and May 2023 where each pen received 12,500 lumpfish only once but not all pens on the same date. Plastic and seaweed shelters were added to pens prior to deployment of lumpfish. The plastic shelters were of the type SeaNest (Imenco, Norway) with a surface area of 120 m2 per shelter. Two shelters were used in each of the pens with plastic shelters. The seaweed shelters were of the type AkvaNest (Tari, Faroe Islands) and the species used was *Saccharina latissima,* which were propagated onto lines in tanks and grown to appropriate size before deployment in the farming pens. Approximately 60 meters of lines with seaweed were deployed in each pen. Two master lines were stretched across the pen diameter with line droppers with seaweed hanging down from the master line every 5 meters.

### Sampling procedure

From the time of lumpfish deployment until the end of the experiment in August various monitoring and sampling took place. Every two weeks, 10 lumpfish from each of the six cages were sacrificed and measured using various welfare parameters as described in Eliasen *et al.* [22]. Sampling for microbiome analyses was performed of lumpfish skin and gills on three occasions using the same individuals that were used for welfare measurements. Microbiome sampling was performed within ~2 weeks following deployment (sampling stage A) and again after ~8 weeks (sampling stage B) and ~14 weeks (sampling stage C). In order to allow sampling to follow this strategy, lumpfish from all pens were not sampled at the same date but at certain intervals following deployment. Lumpfish retrieved from each pen were kept cool and in separate sterile plastic bags until arrival at the field station at the farming site and sampled within an hour. Sampling of skin and gill mucus was performed using sterile swabs prior to welfare measurements. Care was taken to use clean sterile gloves and a sterilized workbench during sampling and sterile plastic tubes for the swabs. The tubes with swabs were immediately stored in dry ice until arrival at the lab where they were stored at –18 °C before DNA extraction within a fortnight. Sampling of the shelters was also performed using swabs. Shelters were raised from the pens and triplicate samples were taken from shelters in each pen at each sampling period. In addition, the shelters were monitored for biofouling each sampling period and categorized from 1 (no biofouling) to 3 (heavy biofouling) in 0.5 increments according to level of biofouling. For seaweed, each sample point was based on 60 measurements of individual leaves, 20 from each of the three pens with seaweed shelters. Contrary to the seaweed shelters, the plastic shelters were a continuous structure and therefore the scoring was performed for each shelter as a whole, two in each of the three pens with plastic shelters. However, at the last sampling stage the scoring of plastic shelters was based on one shelter from each cage due to shortage of manpower at the farming facility. General condition, including tears, holes, and pests for all shelters was estimated using ROV imagery and manual measurements and since categorized in the same manner as biofouling. In addition, size and growth of the seaweed shelters were measured during sampling. An overview of measurements and sampling performed can be seen in Table 1.

**Table 1:** Overview of sampling and measurements.

Stage	Date	Sample origin	Samples and measurements
A	19.04.2023	Lumpfish (n = 10) pen no. 4,12	Microbiome gills & skin, welfare
	03.05.2023	Lumpfish (n = 10) pen no. 1, 3, 7, 8	Microbiome gills & skin, welfare
	22.05.2023	Shelters	Microbiome, biofouling, condition, growth
B	31.05.2023	Lumpfish (n = 10) pen no. 4,12	Microbiome gills & skin, welfare
	20.06.2023	Lumpfish (n = 10) pen no. 1, 3, 7, 8	Microbiome gills & skin, welfare
	20.06.2023	Shelters	Microbiome, biofouling, condition, growth
C	10.07.2023	Lumpfish (n = 10) pen no. 4,12	Microbiome gills & skin, welfare
	26.07.2023	Lumpfish (n = 10) pen no. 1, 3, 7, 8	Microbiome gills & skin, welfare
	07.08.2023	Shelters	Microbiome, biofouling, condition, growth

### DNA extraction, library preparation, and sequencing

DNA extraction was performed using the Soil DNA Isolation Plus Kit (Norgen Biotek, Canada). The lysis buffer from the kit was added to the tubes containing the swabs. The samples were incubated at room temperature for 20 minutes with vortex mixing every 5 minutes. The liquid was then transferred to Bead Tubes from the kit before proceeding with the extraction protocol as described by the manufacturer. The elution volume was 80 ul.

Library preparation was performed using the Quick-16S NGS Library Prep Kit (Zymo Research, Germany) but using the 341f/785r amplicon primers [23,24] and Nextera Index primers (Qiagen, Germany). DNA concentrations were measured using the Qubit dsDNA HS kit (Invitrogen, USA) and Clariostar plate reader (BMG Labtech, Germany). Libraries were sequenced at Novogene (Cambridge, UK) using an Illumina (San Diego, USA) platform producing 2x300 bp paired end reads.

### Data analysis

Raw sequence data were processed using Qiime2 [25] following the methods described in [26]. Once taxonomy, alpha diversity, and beta diversity were obtained, feature tables as well as core metric results and taxonomies were imported into R [27] with “tidyverse” [28] for analysis and plotting using the package “qiime2R” [29]. Data were further processed and filtered for plots using “metacoder” [30]. Unassigned OTUs as well as Eukaryota, Mitochondria, and Chloroplasts were removed to ensure that further analysis was carried out on bacterial communities only.

In order to compare alpha diversity metrics, the following mixed effects linear models were constructed using the package “lme4” [31] with “lmertest” [32]: The difference in diversity between sample types (the tissue type or substrate that the sample originated from: gills, skin, plastic, and seaweed) was analyzed with diversity metrics (Shannon entropy and Faith’s PD) as response variables and sample type as predictor with pen as random factor to control for potential differences between the pens. The effect of shelter type (plastic or seaweed) on diversity on gills and skin was tested with the diversity metrics on those tissue types as response variables with shelter type as predictor again using pen as random factor. Finally, the effect of sampling time stage (A, B, or C) was investigated using diversity metrics in all samples as response variable, sampling stage as predictor, and sample type as random effect to control for the differences between the types of samples.

Differential abundance was tested at genus level using “corncob’s” [33] differentialTest function, which runs a model using maximum likelihood for the Beta-binomial distribution with a logit link function for each taxon. P values were adjusted for multiple testing using the built-in false discovery rate control function. Due to the high diversity, differential abundance analyses were carried out on the top 30 relative most abundant genera to avoid excessively high multiple correction rates. One test was carried out for all samples comparing those from pens with plastic shelters to those with seaweed shelters and controlling for sample type. Subsequent tests compared relative abundance in cages with plastic and seaweed shelters on the sample types separately. Finally differential abundance over time was tested while controlling for sample type and whether samples came from pens with seaweed shelters or plastic shelters.

Differential abundance results are presented in three -part figures. Two bar charts (one for plastic and one for seaweed) showing the relative abundance of the 17 most abundant genera in each group. Additionally, a differential abundance figure presenting the significantly different genera from the differentialTests, showing the expected difference in the logit-transformed relative abundance of genera between the two groups and 95% confidence intervals. Tables show results from significance testing as described above using differentialTest.

Beta diversity analysis was based on a Bray-Curtis dissimilarity matrix and illustrated with a PCoA plot. A permanova test using the Adonis2 function from the package “vegan” [34] was applied to test for potential effects of sample type, sampling stage, and shelter type on beta diversity. Shelter biofouling data were analyzed using a clm from the package “ordinal” [35] with shelter type as predictor and biofouling score as an ordered response variable.

## Results

### Shelters

The estimation of surface area of the seaweed shelters was based on measurements performed at sampling. In May the seaweed leaves were on average 94.6 cm (SD = 23.0) and 8.8 cm (SD = 2.2) in length and width respectively, while in August these values were 137.9 cm (DS = 30.2) and 13.8 cm (SD = 3.1). With approximately 170 individuals per meter of the 60 m line per pen, and adjusting for the shape of the seaweed leaves and non-useable areas by a reduction of 75% and 50%, respectively, the estimated surface area of the seaweed shelters in each pen was between 637 m^2^ to 1,456 m^2^ from deployment to the end of the experiment. In comparison, the plastic shelters had a surface area of 240 m^2^ per pen.

**Fig 2:**
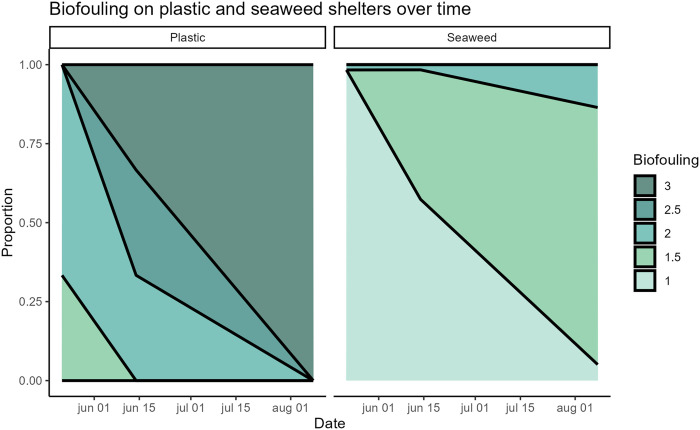
Shelter biofouling. Level of biofouling on seaweed and plastic shelters. Biofouling categories: 1 = no biofouling; 1.5 = sparse biofouling; 2 = full coverage of light biofouling; 2.5 = full coverage of medium biofouling; 3 = full coverage of heavy biofouling.

Based on the biofouling measurements, a cumulative link model showed significant differences between biofouling on the two shelter types (z = −6.151, N = 195, P < 0.001). The plastic shelters had more biofouling than the seaweed shelters at all three measuring periods and had heavy biofouling in all three pens at the last measuring (Fig 2a). The seaweed shelters on the other hand never had more than light biofouling (Fig 2b). Hence, the results from the biofouling measurements suggest that plastic shelters are more prone to biofouling than seaweed shelters, which however can be mitigated by cleaning procedures.

### Sequencing data

From the 414 samples sequenced, the total number of reads after quality filtration and removal of ASVs with less than 10 reads was 311,894,277 with a median of 673,995 reads per sample. One seaweed sample only had 1,811 reads and was discarded for downstream analyses while the second lowest value was 34,994 reads.

Four negative control swabs, opened at various times during field sampling, were processed alongside the other samples. DNA concentrations following DNA extraction were either too low to detect or max 0.5 ng/ul. Following library prep where low concentration samples were subject to more PCR cycles, the DNA concentrations were more similar to those of the other samples. They were sequenced with the other samples and produced on average 503,066 quality filtered reads. The most dominating bacteria in the negative controls were *Methylobacterium* and *Cutibacterium*. This was taken into consideration in the downstream data interpretation.

#### Alpha diversity.

Alpha diversity analysis of the various sample types showed a significant difference in Shannon diversity of the bacterial communities between all sample types (see Table 2 for details).

**Table 2:** Alpha diversity statistics.

Comparison		vs	Shannon entropy	Faith’s PD
Sample types	Gills	Skin	t = 8.07, P < 0.001***	t = 1.38, P = 0.169
		Plastic	t = 17.92, P < 0.001***	t = 2.84, P = 0.005**
		Seaweed	t = 7.11, P < 0.001***	t = −0.03, P = 0.979
	Skin	Plastic	t = 13.92, P < 0.001***	t = 2.15, P = 0.032*
		Seaweed	t = 3.24, P = 0.001**	t = −0.69, P = 0.488
	Seaweed	Plastic	t = 7.61, P < 0.001***	t = 2.08, P = 0.038*
Gills	Seaweed	Plastic	t = −0.86, P = 0.437	t = 3.82, P = 0.019*
Skin	Seaweed	Plastic	t = −1.55, P = 0.196	t = −1.33, P = 0.254
Sampling stage	A	B	t = 5.29, P < 0.001***	t = −0.51, P = 0.608
		C	t = 4.94, P < 0.001***	t = 4.48, P < 0.001***
	B	C	t = −0.33, P = 0.740	t = 4.99, P < 0.001***

Linear mixed effects models comparing alpha diversity metrics (Shannon entropy and Faith’s PD) over sample type (tissue and shelter type), gills and skin over shelter availability in pen, and samples over time. The t and P values are from the summary tables from the models, which provide differences between each factor level. Significance levels: * p < 0.05, ** p < 0.01, *** p < 0.001

**Fig 3:**
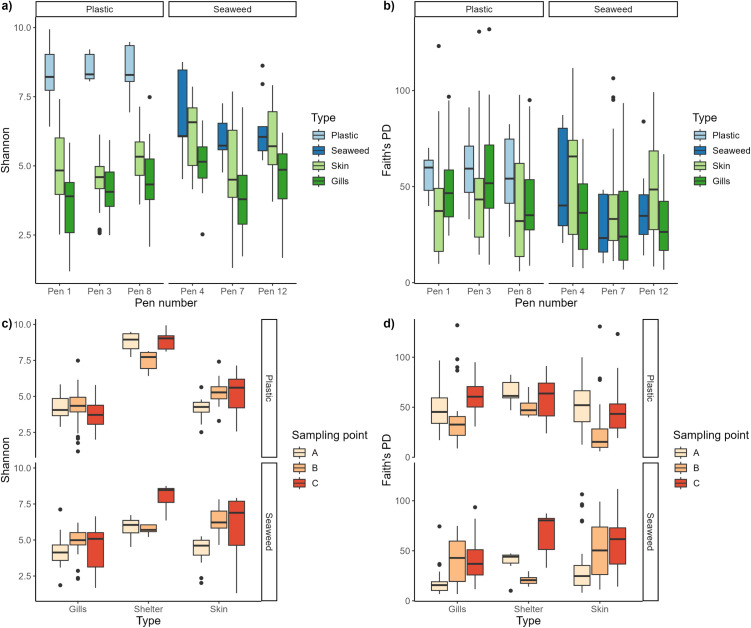
Alpha diversity. Upper row shows alpha diversity measures (a) Shannon and (b) Faith’s PD for all sample types in each pen split by shelter type. Lower row shows (c) Shannon and (d) Faith’s PD alpha diversity measures for each sample type over time split by shelter type.

Plastic shelters had the highest Shannon diversity while gills had the lowest (Fig 3a). Regarding the phylogenetic diversity, only plastic shelters differed significantly from the other sample types (Table 2), having the highest Faith’s PD values (Fig 3b).

The microbial community on gills of lumpfish living in pens with plastic shelters also had a significantly higher phylogenetic diversity than those on gills of lumpfish living in pens with seaweed shelters (t = 3.82; p = 0.019, Fig 3b). In pens with seaweed shelters there was a general trend of increasing Shannon and phylogenetic diversity over the sampling period for all sample types while in pens with plastic shelters the diversity values were more fluctuating (Fig 3cd). The phylogenetic diversity at the last sampling period was significantly different than of the previous two sampling periods, and there was also a significant difference in Shannon diversity between sampling stage A and C (see Table 2). This indicates changes in the microbiome over time although not necessarily the same changes in pens with seaweed and plastic shelters. An overview of the alpha diversity statistical analysis and results can be seen in Table 2.

#### Composition of bacterial communities.

**Fig 4:**
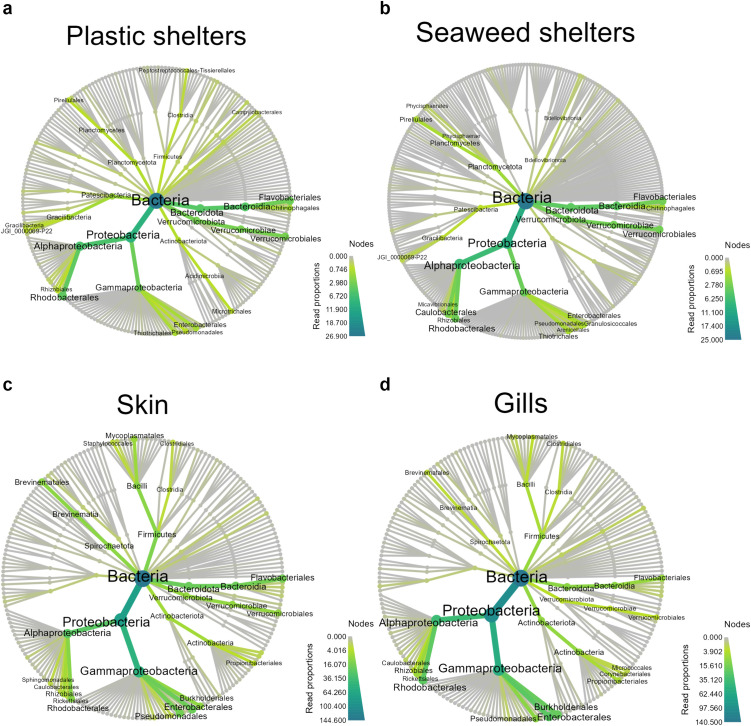
Heat trees. Phylogenetic heat trees down to order level for Bacteria on sample types (a) plastic shelter, (b) seaweed shelters, (c) lumpfish skin, and (d) lumpfish gills.

Proteobacteria was the dominating phyla in all sample types (Fig 4). However, Alphaproteobacteria had higher relative abundance than Gammaproteobacteria in both shelter types while it was the opposite for lumpfish skin and gills. On plastic shelters, Rhodobacterales was the single most dominating order of the Alphaproteobacteria (Fig 4a) while Caulobacterales was equally dominating on seaweed shelters (Fig 4b). On skin and gills, there were several prominent Alphaproteobacteria orders although Rhodobacterales also here was the most dominant order (Fig 4cd).

The orders in Gammaproteobacteria with the highest relative abundance in the lumpfish samples were Enterobacterales, Pseudomonadales, and Burkholderiales (Fig 4cd). Burkholderiales was not prominent on either shelter type, but Thiotrichales was relatively abundant (Fig 4ab). Bacteroidota and Verrucomicrobia were also prominent phyla on the shelters, but less so in the lumpfish samples. Other phyla detected were Planctomycetota and Patescibacteria, mainly in seaweed and plastic shelters respectively (Fig 4ab), Firmicutes and Spirochaetota, mainly in skin samples (fig 4c), and Actinobacteriota in gill samples (Fig 4d).

**Fig 5:**
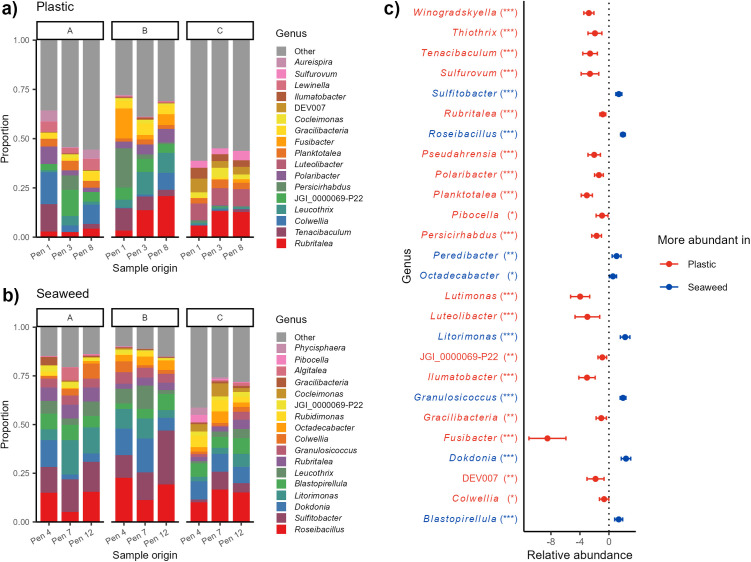
Bacterial communities in shelter biofilm. Bacterial community compositions illustrating the most dominating genera on (a) plastic and (b) seaweed shelters split by pen (x axis) and sampling stage (panel A to C). (c) The genera that were found to be significantly different in abundance between shelter types when analyzing the 30 most abundant genera on the shelters. Red genera (to the left) are more abundant in plastic shelters while blue genera (to the right) are more abundant on seaweed shelters. Significance levels: * p < 0.05, ** p < 0.01, *** p < 0.001.

Almost half of the bacteria detected on the shelters were exclusively found on plastic (25.7%) or seaweed (18.8%) shelters, indicating quite different bacterial communities on the two shelter types. The differences between the bacterial communities on plastic and seaweed shelters were also apparent when looking at the most dominant bacteria, as these were not the same on seaweed and plastic shelters. Less than half of the most relative abundant genera were so on both shelter types (Fig 5ab).

The top 17 dominating bacterial genera on the plastic shelters consisted of a lower percentage of the total reads than those on seaweed shelters, aligning with the higher Shannon diversity detected in the plastic samples. Among the dominating bacteria found on both shelter types were mostly well-known marine genera such as *Polaribacter*, *Sulfitobacter*, and *Colwellia* that are widely distributed, especially in the colder regions [36–38]. *Rubritalea* was the most dominating genus on plastic shelters and *Fusibacter*, which is mostly known for containing various pathogens affecting humans and some terrestrial animals, was also among the prominent bacteria on plastic shelters. Recently, however, a study detected an aero-, halo-, and psychrotolerant *Fusibacter* strain inhabiting the marine sediment in an arctic region, suggesting that *Fusibacter* could be more widely distributed than previously thought [39].

Overall, there were similar patterns of dominating bacteria on the same shelter types in the various pens, but some changes were apparent over time, especially on plastic shelters (Fig 5ab). Statistical comparisons of the bacterial community compositions on the two shelter types showed a significant difference in the majority of the 30 genera with highest relative abundance (Fig 5c). Among these genera was the pathogenic genus *Tenacibaculum,* which was the second most abundant genus detected on plastic shelters but was not identified among the dominant bacteria on seaweed shelters (Fig 5ab). Among the genera with significantly higher relative abundances on seaweed shelters were *Dokdonia* and *Blastopirellula,* previously detected as part of the biofilm forming bacterial communities on various macroalgae [40–42].

**Fig 6:**
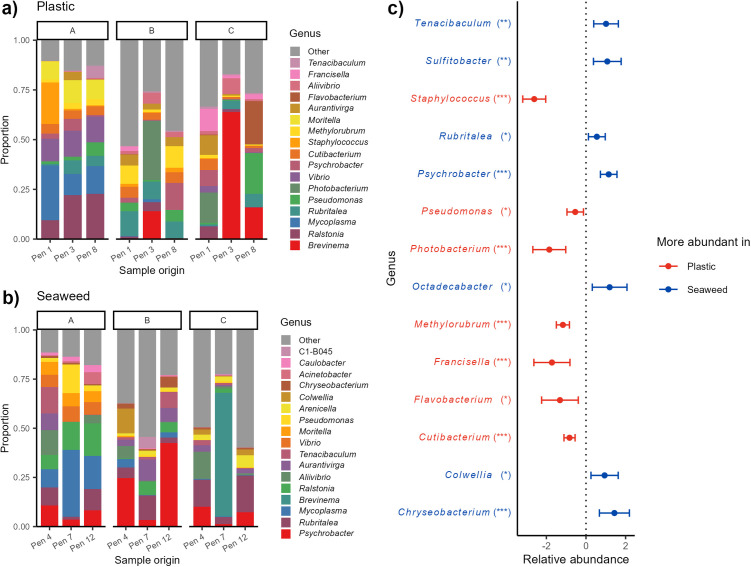
Bacterial communities in lumpfish skin mucus. Bacterial community compositions illustrating the most dominating genera on lumpfish skin from pens with (a) plastic and (b) seaweed shelters split by pen (x axis) and sampling stage (panel A to C). (c) The genera that were found to be significantly different in relative abundance on lumpfish skin depending on shelter type when analyzing the 30 most relative abundant genera on skin from all pens. Red genera (to the left) have higher relative abundances on lumpfish in pens with plastic shelters while blue genera (to the right) have higher relative abundances on lumpfish in pens with seaweed shelters. Significance levels: * p < 0.05, ** p < 0.01, *** p < 0.001.

Analysis of bacterial communities in the lumpfish skin microbiome showed a change over time in dominance of the major groups. This shift was apparent in the skin of lumpfish living in pens with both plastic and seaweed shelters (Fig 6ab). The lumpfish from the triplicate pens with the same shelter type also seemed to have more similar bacterial community compositions early after deployment and differ more at the other two sampling stages (Fig 6ab). In the top 30 most relatively abundant genera, fourteen genera differed significantly in relative abundance between the skin of lumpfish in pens with seaweed shelters and those with plastic shelters (fig 6c).

The eleven relatively most abundant bacterial genera in the skin microbiome of lumpfish from pens with seaweed shelters were also among the 17 genera of highest relative abundance on lumpfish from pens with plastic shelters (Fig. 6ab). The genera *Tenacibaculum*, *Vibrio, Moritella, Pseudomonas* and *Aliivibrio* with known pathogenic species [43,44] were detected among the most dominant genera on lumpfish from pens with both shelter types. However, *Tenacibaculum, Moritella* and *Vibrio* were mainly present shortly after the lumpfish were deployed and were much less dominant at the second and third sampling stages (Fig 6ab). In addition to these, several other potential pathogens such as *Francisella* [45]*, Staphylococcus* [46]*, Flavobacterium* [47] were also detected among the dominant genera in skin samples from lumpfish living in pens with plastic shelters (Fig 6b). These four potentially harmful genera all had significantly higher relative abundance on lumpfish from pens with plastic shelters, while *Tenacibaculum* was significantly more abundant on lumpfish from pens with seaweed shelters (Fig 6c). However, the potentially beneficial bacteria *Psychrobacter* [48] was the most dominant genus in skin samples from lumpfish living in pens with seaweed shelters. In contrast, lumpfish living in pens with plastic shelters had significantly lower relative abundance of *Psychrobacter* in the skin microbiome. At the last sampling period, the genus *Brevinema* was also highly noticeable in the skin samples of lumpfish from pens 7 and 3 containing seaweed and plastic shelters respectively.

**Fig 7:**
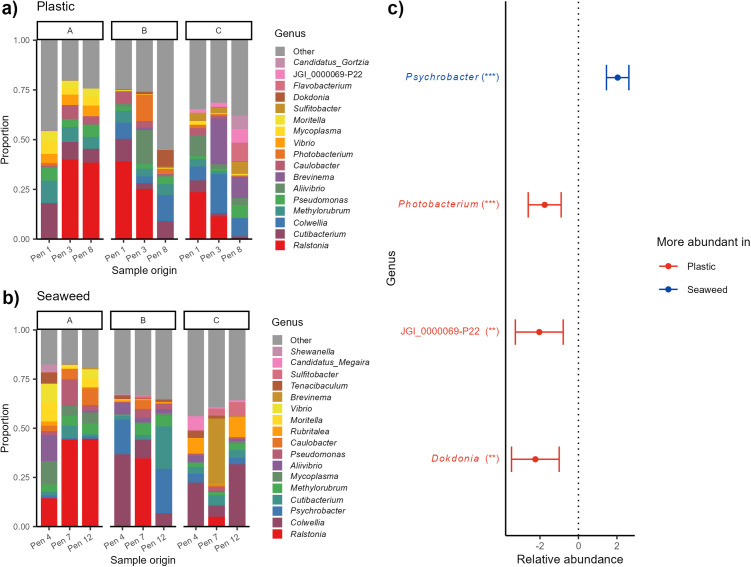
Bacterial communities in lumpfish gill mucus. Bacterial composition of the most dominating genera in lumpfish gills in (a) seaweed and (b) plastic shelters split by pen (x axis) and sampling stage (panel A to C). (c) The genera that were found to be significantly different in abundance on lumpfish gills depending on shelter type when analyzing the 30 most abundant genera on lumpfish gills from all pens. Red genera (to the left) have higher relative abundance on lumpfish in pens with plastic shelters while blue genera (to the right) have higher relative abundance on lumpfish in pens with seaweed shelters. Significance levels: * p < 0.05, ** p < 0.01, *** p < 0.001.

Many of the dominant genera detected in skin samples were also among the dominant genera in the gill samples (Fig 7ab). *Ralstonia*, which was among the dominating genera in skin samples, was the genus with the highest relative abundance in gills of lumpfish living with both shelter types.

Several of the dominating genera detected in lumpfish skin (Fig 6ab) were also among the dominating genera in lumpfish gills (Fig 7ab). In addition, twelve of the seventeen most dominating genera in the gill microbiome of lumpfish in pens with seaweed and plastic shelters were the same (Fig 7ab), similar to the pattern seen in skin samples. However, bacterial communities in the gill microbiomes of lumpfish living in pens with seaweed and plastic shelters were more similar than what was the case for bacterial communities in the skin microbiomes, as only four genera among the top 30 most dominating genera were significantly different (Fig 7c). The only genus of the four with significantly different relative abundance that was more prominent in lumpfish living in pens with seaweed shelters was *Psychrobacter.* On the other hand, one of the three genera with significantly higher relative abundance in skin of lumpfish living in pens with plastic shelters was *Photobacterium*, which contain species that can be pathogenic in warmer waters [49]. Other dominating genera were well known marine bacteria such as *Colwellia*, which is widespread in the colder marine regions [50], *Cutibacterium* commonly found in the gut microbiome and sometimes gills of marine fish [51,52] and *Mycoplasma* that often is a dominant part of the bacterial communities in salmon gut microbiome [53,54].

#### Beta diversity.

**Fig 8:**
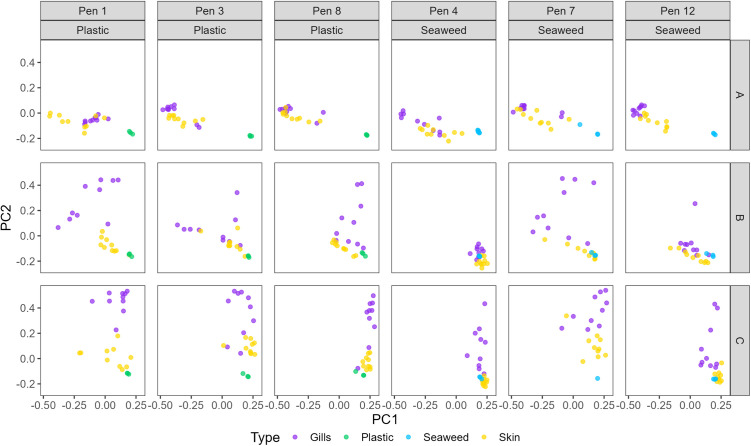
Bray Curtis PCoA. PCoA plot based on Bray Curtis beta diversity distance matrix. Samples are split into columns by pen, with those containing plastic shelters to the left and those with seaweed shelters to the right. The rows are the three sampling stages A, B, and C. Colors represent sample types.

The beta diversity analysis, illustrated in a Bray Curtis PCoA plot (Fig 8), showed a change occurring over the experimental period. Skin and gill samples in yellow and purple, respectively, were partially intertwined and/or clustered near each other early after deployment. Over time the variation described changed from PC1 to PC2 and the two sample types had a little less overlap. On the other hand, the shelter samples were separated from the lumpfish samples at the first sampling stage, but over time the skin samples separated somewhat from the gill samples and drew closer to the shelter samples. The bacterial communities on the gills seemed less affected by the shelters as the gill samples were distanced further away from the shelter samples.

A permanova test investigating potential effects of sample type, sampling stage, and shelter type on the Bray Curtis distance matrix was performed. All three parameters had a significant impact on distances between samples (sample type: F_2,409_ = 11.873, p < 0.001; sampling stage: F_2,409_ = 11.518, p < 0.001; shelter type: F_1,409_ = 2.631, p < 0.001). The statistical analyses support what the PCoA plot illustrated (Fig 8), that there was a more visible difference between the sample types and sampling stages than between skin and gill samples from lumpfish living in pens with either plastic or seaweed shelters. However, there still was a significant difference between the bacterial communities in the microbiome of lumpfish living with the two shelter types.

### Welfare indicators

**Fig 9:**
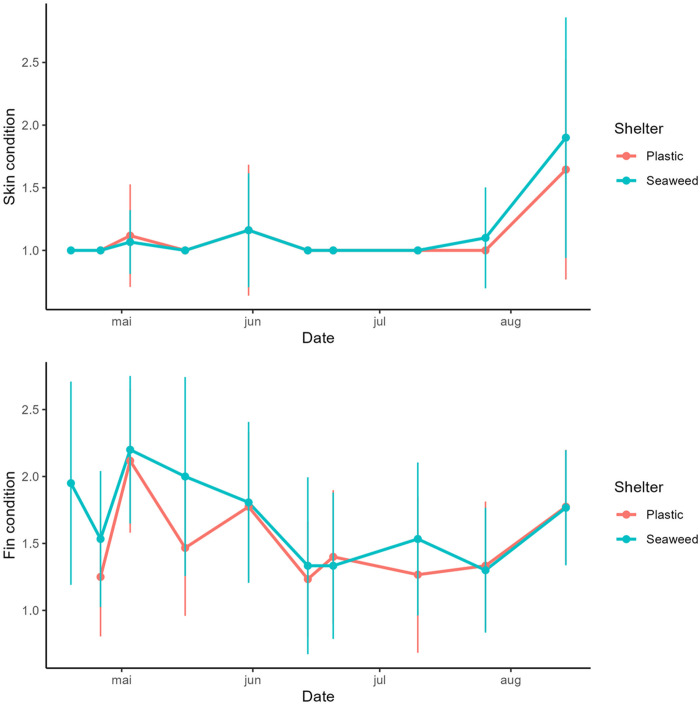
Welfare indicators. Measurements of lumpfish condition. The upper plot shows skin condition and the lower plot shows fin condition. Points are average and whiskers are standard deviation of possible categories 1, 2, and 3 representing good medium and bad condition, respectively.

The results from the welfare measurements including fin and skin condition, characterized 1–3 representing good to bad, showed overall low indicator values for the skin (Fig 9a), meaning the lumpfish were in good condition, while the fin condition was generally higher (Fig 9b).

No significant differences in fin and skin condition were detected between lumpfish in pens with plastic and seaweed shelters. Hence, any differences in the relative load of potentially harmful bacteria in the skin mucus were not reflected in the welfare indicators of the lumpfish at the time of sampling.

## Discussion

Ocean based aquaculture facilities are built environments and as such they have distinct microbiomes associated with the various structures and materials [18]. Farmed salmon living in these large constructed environments might have limited contact with the structures themselves, while lumpfish that rely more on shelter invariably are in more frequent contact with these surfaces. It is critical for the welfare of lumpfish used as cleaner fish in the aquaculture industry that shelters are placed in the pens [55], and that these shelters are suitable and do not cause unintended harm. The plastic shelters investigated in this project were more prone to biofouling than the seaweed shelters used. This suggested that the seaweed shelters were a better environment for the lumpfish that were in regular close contact with the shelters. However, biofouling on the plastic shelters can be mitigated with cleaning procedures, although it might cause some disturbance for the lumpfish. Bacterial communities in the biofilm of both plastic and seaweed shelters had significantly higher Shannon diversities than the bacterial communities in lumpfish skin and gill microbiomes, but the Shannon diversity of the bacterial communities on plastic shelters were also significantly higher than those on the seaweed shelters. The plastic shelter microbiome also had higher phylogenetic diversity than that of the seaweed shelters, indicating that fewer bacterial taxa could thrive on live *Saccharina latissima* leaves, used as seaweed shelter in this case, than on the plastic shelters. Another sign of the difference between the shelter types was that only about half of the bacterial genera detected on the shelters were on both shelter types while the others were solely found on either plastic or seaweed shelters. This was reflected in the high number of significant differences in the relative content of various bacteria. Among these, a significant difference was detected between the relative abundances of *Tenacibaculum* on the two shelter types. However, the relative abundance of *Tenacibaculum* diminished over time and this pattern was also observed in some of the lumpfish skin samples. Earlier investigations have detected high loads of *Tenacibaculum* in the transport water and in samples from lumpfish being shipped from abroad to the Faroe Islands for use as cleaner fish in the aquaculture industry (pers. com. Esbern J. Patursson). Therefore, it might be possible that these bacteria were already part of the lumpfish microbiome when transported to the aquaculture farming site, though this is only speculation. Although the relative abundance of *Tenacibaculum* diminished over time on the plastic shelters, it might be noteworthy that they were never among the dominating genera on the seaweed shelters. This finding is consistent with the results from a study by Liu *et al*. [56] investigating biofilm on cultured *Saccharina latissima* where they detected a much lower relative abundance of *Tenacibaculum* on the seaweed than in the surrounding seawater. Others have also detected a relatively high load of potentially harmful bacteria on plastic in marine aquaculture operations, while the bacterial composition differs from the surrounding seawater [57,58]. Although no seawater sample was included in this study, the aquaculture site chosen for the experiment was placed in an area with good water exchange providing the best possibility for similar conditions for all pens. The most dominating genus on plastic shelters was *Rubritalea*, consistent with the findings of Papale *et al*. [59] who consistently found *Rubritalea* in biofilm on plastic submerged in seawater. Other genera with significantly higher relative abundance on plastic shelters included the marine genus *Planktotalea*, which contains only a few documented species detected in colder marine regions [60,61]. However, *Planktotalea* was noticeably detected in plastic biofilm and not the surrounding seawater in the study by Papale *et al*. [59]. Overall, the data indicated that the two shelter types provided different environments for the lumpfish in this study.

The gill samples of lumpfish living with either shelter type had overall more similar bacterial communities than the other sample types. This suggested a higher reflection of the surrounding seawater, which in a farming facility with good water exchange, such as Froðba [21], should have a relatively homogenous mixture of the microbial community coming through the various pens. Other studies have also found that gills had the highest similarity with seawater although there were tissue specific microbial communities on fish [52]. Many of the dominating genera present in the gill samples were also well-known ubiquitous marine bacteria or fish gut bacteria. However, we find it interesting that of the 30 most dominating genera only *Psychrobacter* had significantly higher relative abundance in gills of lumpfish from pens with seaweed shelters. *Psychrobacter* is often used as a probiotic and has been demonstrated to reduce the negative effects of *Tenacibaculum* in fish [48]. *Psychrobacter* was also the genus of highest relative abundance in skin samples from lumpfish living with seaweed shelters and their relative abundance in the skin from lumpfish living in pens with plastic shelters was significantly lower. This might suggest a beneficial effect from the seaweed shelters, although *Psychrobacter* was not detected among the dominating bacteria on the seaweed itself.

The skin samples contained overall many potentially harmful bacteria among the dominating genera. Lumpfish from pens with plastic shelters had a higher number of these and with a combined higher relative abundance. In addition, for many of the dominating and potentially harmful bacteria detected on lumpfish from pens with seaweed shelters the relative abundance was high shortly after deployment and diminished throughout the sampling period. Only *Aliivibrio* was relatively abundant in one of the pens with seaweed shelters at the final sampling.

In pens with plastic shelters, there was also a shift in microbial composition on the lumpfish skin. *Brevinema*, which has been associated with the gut microbiome of several fish species, including salmon [62], had a high relative abundance in pens with both shelter types at the last sampling. The presence of *Brevinema* on the lumpfish skin is likely a reflection of the salmon feces in the water and the genus has not been reported to include pathogens. On the other hand, there were still several potentially harmful bacteria among the dominant genera in the lumpfish skin in pens with plastic shelters at the final sampling period, e.g., *Pseudomonas, Francisella, Aliivibrio and Flavobacterium*. Combined, these observations suggested that lumpfish living with seaweed shelters had healthier skin microbiome. However, the welfare measurements did not indicate any differences between lumpfish living in pens with plastic or seaweed shelters. The lumpfish were generally in good condition, and it can only be speculated whether more stressful conditions would have resulted in significantly lower welfare for lumpfish in pens with plastic shelters than with seaweed shelters. Also, there is evidence from the literature that the commensal bacterial community on fish is species specific [63,64] and that commensal bacteria can also become opportunistic pathogens [65]. Together, these traits make it difficult to determine precisely which bacteria are harmful, commensal or perhaps beneficial at any given time for a specific fish species. However, any condition that might cause a clear dominance of potentially harmful bacteria in the skin mucus of lumpfish used as cleaner fish should be kept in mind as other factors weakening the fish might reveal correlations not previously detected.

The beta diversity analysis showed that over time the bacterial communities in microbiomes of the lumpfish skin and the shelters became more similar while the gill samples became more separated from the skin samples and to a lesser extent drew closer to the shelters. The most prominent genera in the negative controls were also among prominent genera in the gill samples from lumpfish living with both shelter types. Since these genera are widely distributed in the marine environment, it could be misleading to disregard these genera due to their presence in the negative controls. We consider the eDNA detected in the negative controls to most likely be an effect of the miniscule amounts of saltwater in the air where negative control tubes were opened during fieldwork. Although the joint water environment likely had some influence on the increased similarity between the lumpfish and shelter microbiome over time, there was a strong indication that especially the skin microbiome was affected by the shelter the lumpfish was living in, correlating with the significant difference between microbial compositions on the skin of lumpfish living in plastic shelters and those living in seaweed shelters.

## Conclusions

The results from this study indicated that plastic and seaweed shelters provided different environments for lumpfish residing amongst them and affected their skin microbiome. Furthermore, bacterial communities in the skin microbiome of lumpfish living in pens with plastic shelters contained more potentially harmful bacteria among the dominating genera than those in the skin microbiome of lumpfish living in pens with seaweed shelters. However, no significant difference was detected in the welfare indicators of the lumpfish, which generally were in good condition. Further investigations are needed to determine whether or not lumpfish under more stressful conditions are more vulnerable to infections when bacterial communities on their skin are dominated by potentially harmful bacteria.

## Supporting information

S1 Data Seawater temperature and oxygen levels during study period. (XLSX)

S2 Data Shelter data. (CSV)

S3 Data Welfare data. (XLSX)
